# A hemolysin secretion pathway-based novel secretory expression platform for efficient manufacturing of tag peptides and anti-microbial peptides in *Escherichia coli*

**DOI:** 10.1186/s40643-021-00471-6

**Published:** 2021-11-26

**Authors:** Wen Zhu, Lifu Hu, Yang Wang, Liangyin Lv, Hui Wang, Wenqiang Shi, Jianwei Zhu, Huili Lu

**Affiliations:** Engineering Research Center of Cell and Therapeutic Antibody, Ministry of Education, School of Pharmacy, Shanghai Jiao Tong University, 800 Dongchuan Road, Shanghai, 200240 China

**Keywords:** *E. coli*, Hemolysin A, Secretory expression, Tag peptides, Anti-microbial peptides

## Abstract

**Background:**

Although *Escherichia coli* has been widely used for the expression of exogenous proteins, the secretory expression in this system is still a big obstacle. As one of the most important secretion pathways, hemolysin A (HlyA) system of *E. coli* can transport substrates directly from the cytoplasm to extracellular medium without the formation of any periplasmic intermediate, making it an ideal candidate for the development of the secretory production platform for exogenous proteins.

**Results:**

In this work, we developed a novel production platform, THHly, based on the HlyA secretion system, and explored its applications in the efficient preparation and quick detection of tag peptides and anti-microbial peptides. In this novel platform the signal sequence of HlyA is fused to the C-terminal of target peptide, with Tobacco Etch Virus (TEV) protease cleavage site and 6*His tag between them. Five tag peptides displayed good secretory properties in *E. coli* BL21 (DE3), among which T7 tag and S tag were obtained by two rounds of purification steps and TEV cleavage, and maintained their intrinsic immunogenicity. Furthermore, Cecropin A and Melittin, two different types of widely explored anti-microbial peptides, were produced likewise and verified to possess anti-microbial/anti-tumor bioactivities. No significant bacterial growth inhibition was observed during the fusion protein expression, indicating that the fusion form not only mediated the secretion but also decreased the toxicity of anti-microbial peptides (AMPs) to the host bacteria. To the best of our knowledge, this is the first report to achieve the secretory expression of these two AMPs in *E. coli* with considerable potential for manufacturing and industrialization purposes.

**Conclusions:**

The results demonstrate that the HlyA based novel production platform of *E. coli* allowed the efficient secretory production and purification of peptides, thus suggesting a promising strategy for the industrialized production of peptide pharmaceuticals or reagents.

**Graphical Abstract:**

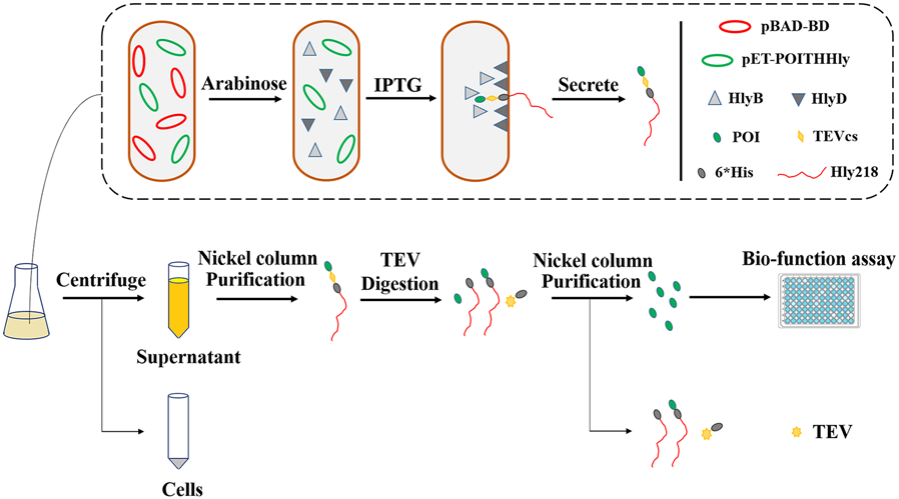

**Supplementary Information:**

The online version contains supplementary material available at 10.1186/s40643-021-00471-6.

## Background

The production of recombinant proteins in microbial systems has revolutionized both fundamental research and industrial production. Among the numerous expression systems, *E. coli* has established itself as one of the most excellent, owing to its advantages of fast and easy transformation with exogenous DNA, low-cost medium, and unparalleled growth rate which boosts rapid protein expression in a short fermentation period (Hayat et al. 2018). However, some problems to this system exist, such as the low yield caused by the degradation by proteases (Kaur et al. 2018) or the intrinsic short half-life of the protein of interest (POI), the toxicity of accumulated POI toward the host cell, and the formation of inclusion bodies which require tedious denaturation and refolding procedures (Park et al. 2018; Carmignotto and Azzoni 2019; Xi et al. 2017). To improve the product solubility, stability, and biological activity, as well as to simplify the downstream purification, a feasible and efficient secretory *E. coli* expression system has long been desired.

There are five known secretion systems (types I through V) for protein translocation across the membrane in *E. coli* (Burdette et al. 2018), among which the type I secretion system (T1SS) secretes toxins and exo-enzymes via a one-step process across both the cytoplasmic and outer membranes without any periplasmic intermediate (Spitz et al. 2019). Given the considerable advantage of one-step secretion compared to other widely applied secretion signal peptides, such as Sec B, a great deal of efforts have been made to establish T1SS for recombinant protein production, especially the use of hemolysin A (HlyA) T1SS (Thomas et al. 2014a, b; Ruano-Gallego et al. 2019; Khosa et al. 2017).

Hemolysin A, a 110 kilo-Dalton (kDa) toxin, secreted from its C-terminus in liner structure, folded and activated into a fully functional pore-forming toxin (Nicaud et al. 1986). Heterologous protein fused to the HlyA signal sequence (C-terminal 218 amino acids) can be recognized and secreted with the help of other three accessory components: HlyB (an ATP-binding cassette (ABC) protein), HlyD (a membrane fusion protein) and TolC (an outer membrane protein) (Kanonenberg et al. 2018). Gene HlyA, HlyB, and HlyD are absent in the genome of prototypical laboratory strains, whereas TolC is encoded in a different location of the chromosome and thus maintained. There are multiple studies which secreted proteins in a functional form using C-terminal end of HlyA, including the β-lactamase (Chervaux et al. 1995), streptokinase (Kern and Cegłowski 1995), maltose-binding protein (Bakkes et al. 2010), alkaline phosphatase (Angkawidjaja et al. 2006), mammalian fatty acid-binding protein (Schwarz et al. 2012a, b), a single-chain Fv antibody (Fernández et al. 2000), and nanobodies (Ruano-Gallego et al. 2019; Fraile et al. 2004). However, all the previous studies were limited to one certain target, and the potential of this secretion system is still to be exploited for generalized applications.

Tag peptides, which comprise several to dozens of amino acids, make protein expression, detection, and identification very robust and easy (Hou et al. 2012). Different tag peptides available nowadays are suitable for a wide range of applications including Enzyme Linked Immunosorbent Assay (ELISA), Immuno-precipitation (IP), Western Blot (WB), Immunofluorescence Microscopy (IFM), and protein purification (Wakasa et al. 2020). Over recent years, these tag peptides have been produced by direct chemical synthesis. However, the low efficiency, low yield, and cost-consuming technology have impeded their large-scale production.

Anti-microbial peptides (AMPs), a large and diverse family of peptides forming part of the innate immune system in nearly all living organisms, are triggering growing attention. With rapid and broad-spectrum activity against bacteria, fungi, viruses, and some parasites, AMPs have been regarded as promising drug candidates to overcome bacterial resistance to currently prescribed antibiotics (Mahlapuu et al. 2016; Greber and Dawgul 2017). Naturally occurring AMPs are generally hydrophobic, positively charged, and comprised of 20–60 amino acid residues with various structures, such as α-helix, β-sheet, and extended or loop-like structures (Fry 2018). The killing mechanisms of AMPs are thought to be diverse, including the formation of transient transmembrane pores, inhibition of cell wall synthesis or other enzymatic activities, and inhibition of essential intracellular proteins (Bechinger and Gorr 2017; Nuti et al. 2017). Moreover, several AMPs also exhibit anticancer activity or secondary functions in immunomodulation, which has further stimulated interest in medicinal study (Otvos 2016). However, preclinical studies require great amounts that cannot be produced in a cost-effective manner by chemical synthesis, and recombinant expression is, therefore, necessary. Cecropin A, a linear cationic α-helical AMP isolated from the moth *Hyalophora cecropia*, shows strong antimicrobial activities against both Gram-negative and Gram-positive bacteria, and does not induce erythrocytes or lymphocyte lysis, even at high concentrations (Agrawal and Weisshaar 2018; Sang et al. 2017). It has been expressed in *E. coli* by conjoining His tag (Xu et al. 2007), His tag-SUMO (Wei et al. 2018), or a self-assembling peptide (ELK16) (Wang et al. 2018, 2019). Melittin, the major component of honey bee venom with 26 amino acids, where the N-terminal 20 amino acids are hydrophobic, while the left 6 ones are positive-charged (Memariani et al. 2019), has been expressed in *E. coli* fused with GST tag (Chen et al. 2021). However, up to now, almost all AMPs produced in *E. coli* are intracellular, which means that the bacteria cell disruption and tedious purification procedures are inevitable. In addition, proteolytic degradation of peptides and antimicrobial toxicity toward the host are also tricky problems. From this point, it is meaningful to develop a suitable system to facilitate the secretory expression of AMPs.

In this study, we set up a novel secretory expression system named ‘THHly’ employing the C terminal end of HlyA. Using this system, five tag peptides were verified to achieve efficient secretory expression and the yield of the fusion proteins was up to 337 mg/L. The immunogenicity assays approved that this secretion system can be utilized to produce peptides or detect the binding property of an antibody to a liner epitope efficiently. Cecropin A and Melittin were also secreted and purified by the secretory system and the products were characterized to possess anti-microbial/anti-tumor activities. Based on these results, the hemolysin secretion pathway-based novel platform THHly provides a reliable source for the efficient production and easy purification of peptides, which enlightens their industrial manufacturing, as well as inspires the efficient secretory production of biopharmaceutical molecules.

## Results and discussion

### Construction and verification of the THHly platform

**Fig. 1:**
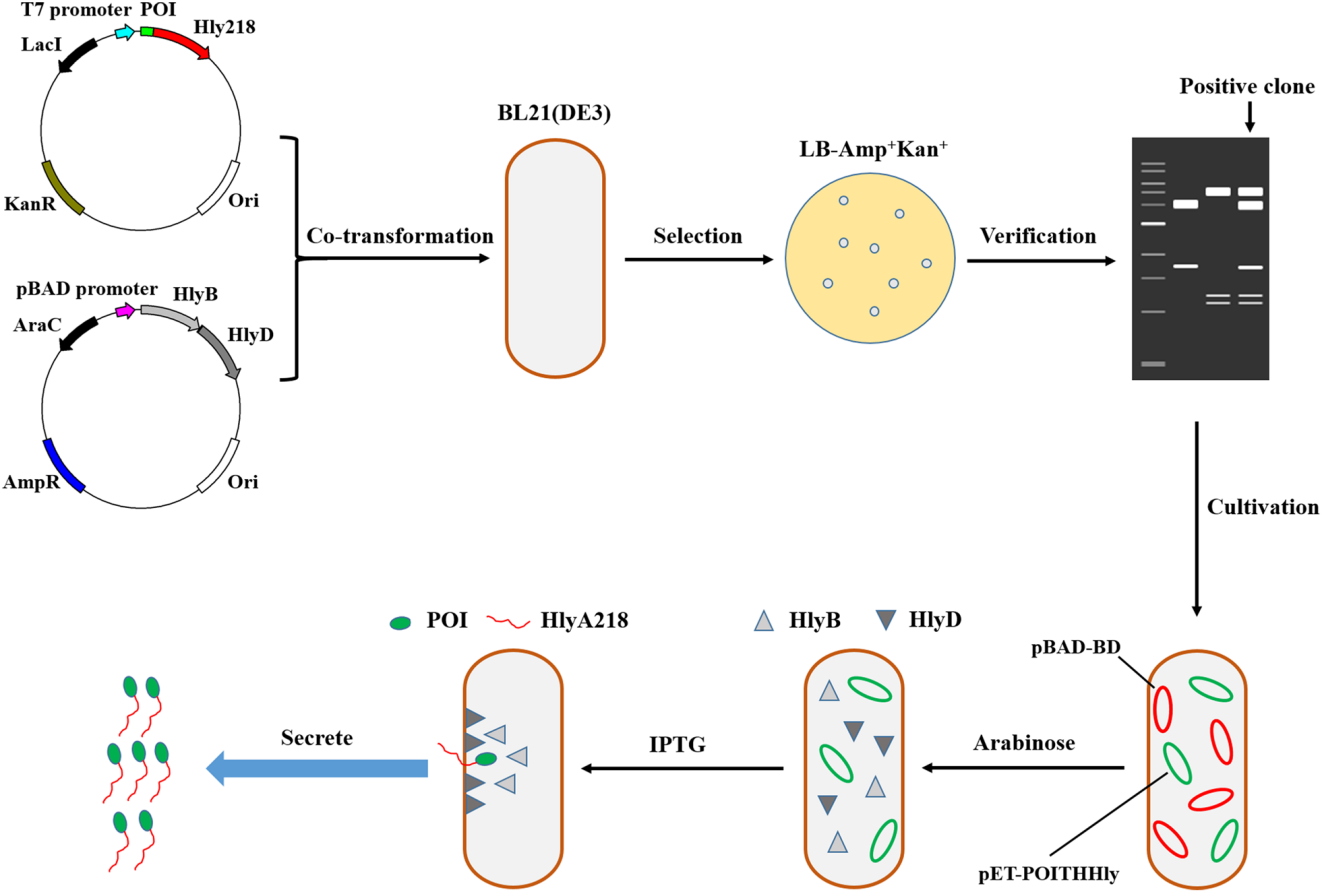
Scheme of plasmids construction, expression strains selection and protein secretion. The expression plasmid and accessory plasmid were co-transformed into BL21 (DE3) and selected by LB-Amp + Kan agar plate. Under the sequent induction of Arabinose and IPTG, the POI were secreted to supernatant

The fusion construction of the THHly platform is composed of four essential elements: POI, Tobacco Etch Virus (TEV) protease cleavage site (T), 6*His tag (H), and Hly218 which consists of the last 218 amino acids of HlyA (Hly), that is, the POI-THHly. The scheme for plasmids construction and expression strains selection is shown in Fig. 1. Briefly, after co-transformation of expression plasmid and accessory plasmid, the expression strains which harbored the two plasmids were selected by LB agar plate containing Ampicillin and Kanamycin. Arabinose (Ara) and isopropyl-1-thio-β-d-galactoside (IPTG) were added in sequence so the POI-THHly was secreted to the supernatant with the help of the accessory proteins.

**Fig. 2:**
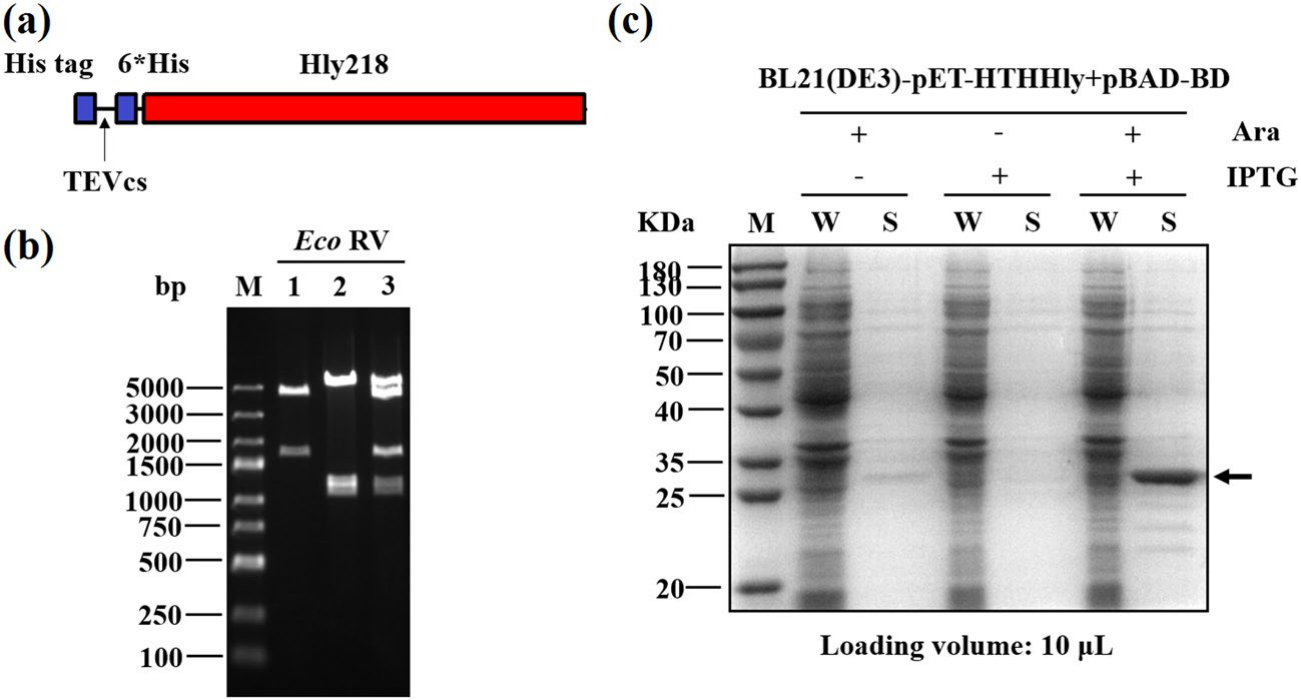
Secretory expression of HTHHly. **a** Plasmid pET-HTHHly. **b** Verification of the expression strain harboring the two plasmids. Lane 1: plasmid pET-HTHHly; Lane 2: plasmid pBAD-BD; Lane 3: plasmid extracted from BL21(DE3)-pET-HTHHly + pBAD-BD. **c** Secretory expression of HTHHly. *W* whole-cell lysate, *S* supernatant. The arrow indicates the target protein HTHHly

To verify the secretion mediated by the HIy system, His tag was utilized as the POI to generate the fusion construct HTHHIy for this purpose (Fig. 2a). The clones on the LB agar plate were picked and proved to harbor the two plasmids (Fig. 2b). A predominant band was observed in SDS-PAGE after induction with Ara and IPTG (Fig. 2c), which matches the theoretical molecular weight of the HTHHly fusion protein calculated from the amino acid sequence (26.9 kDa), indicating that the fusion protein was successfully secreted at a high level. Notably, no corresponding band was seen in the supernatant or the cell pellet of the Ara^−^IPTG^+^ group. We speculate that the target protein is subject to protease degradation inside the bacteria, which suggests the great benefit of secretory expression. With the addition of arabinose, a tiny amount of HTHHly was secreted for the background promotion of T7 promoter (Group Ara^+^IPTG^−^).

### Optimization of the secretory expression conditions

**Fig. 3:**
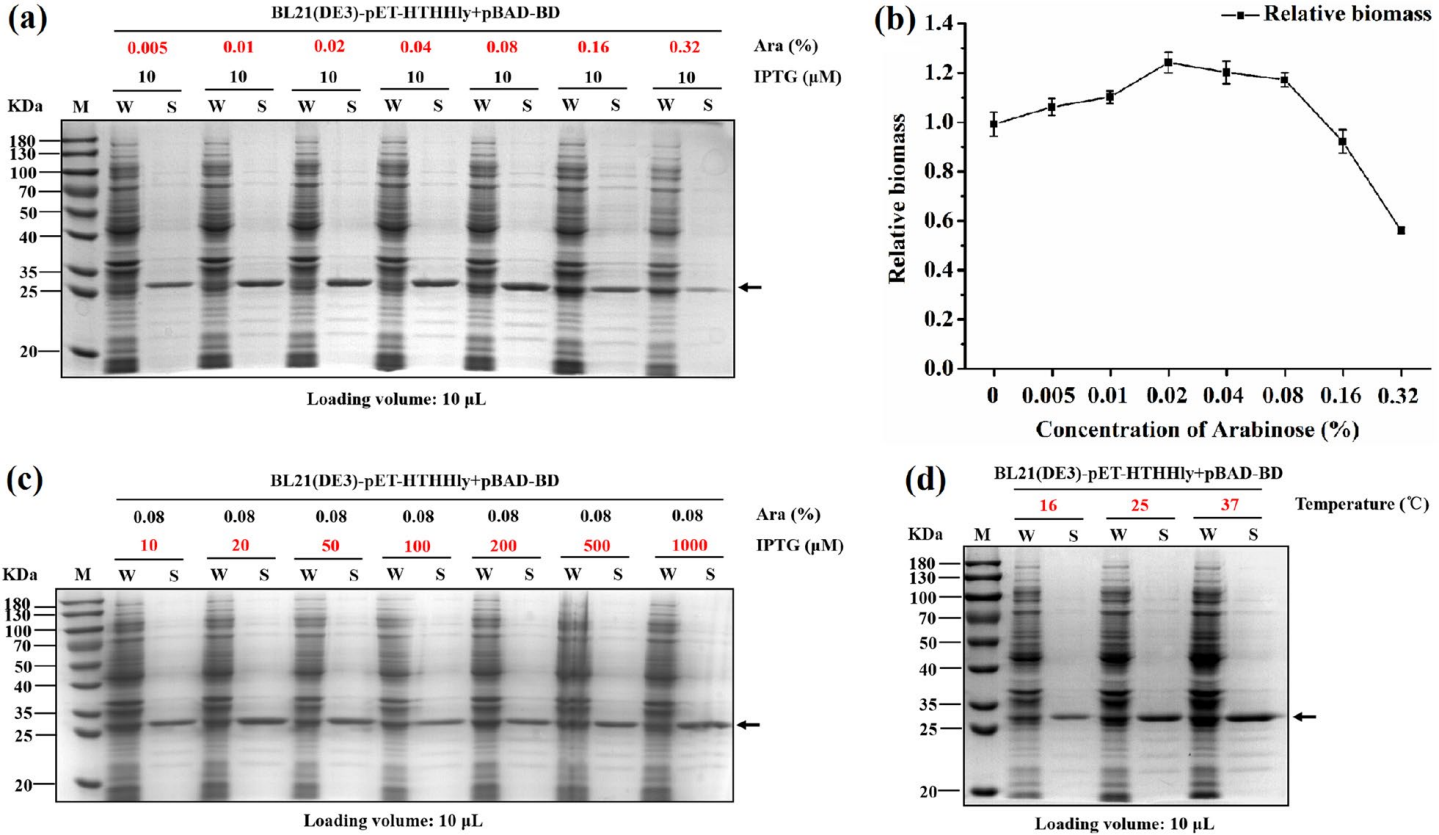
Optimization of the Hly secretion system with HTHHly. **a** Effect of arabinose concentrations on secretion level. **b** Effect of arabinose concentrations on biomass. **c** Effect of IPTG concentrations on secretion level. **d** Effect of induction temperatures on secretion level. *W* whole-cell lysate, *S* supernatant. The arrows indicate the target protein HTHHly

To improve the secretory expression level of the fusion protein, different concentrations of Ara (0.005–0.32%), IPTG (10–1000 μM), and induction temperatures (16, 25, and 37 °C) were investigated. The secretion level increased along with the increase of arabinose concentrations but declined sharply when it was above 0.08% (Fig. 3a). The cultures were sampled, diluted, and detected for the OD_600_ absorbance. From the cell biomass we infer that an overdose of arabinose led to a massive expression of HlyB and HlyD, thus playing an adverse impact on cell growth (Fig. 3b). The concentration of IPTG in the studied range seemed to be insignificant (Fig. 3c). The secretory level increased significantly at higher induction temperature (Fig. 3d). As one type of ABC transporter in *E. coli*, the Hly secretory system is an energy-dependent efflux transporter and susceptible to the ATP level (Kanonenberg et al. 2019). Induction at physiological-preferred temperature could help the cells maintain a vigorous metabolic state and keep the Hly secretory system work efficiently. It has been demonstrated that the addition of Ca^2+^ improved the secretion of the Hly system based on the hypothesis that binding of Ca^2+^ to the GG repeats triggers folding of HlyA in the extracellular space (Thomas et al. 2014a, b), but it didn’t work in this study (data not shown). In summary, 0.08% Ara, 100 μM IPTG, and 37 °C were optimal and selected as the induction conditions for the expression of target proteins.

### Secretory expression and purification of the tags fusion protein

**Fig. 4:**
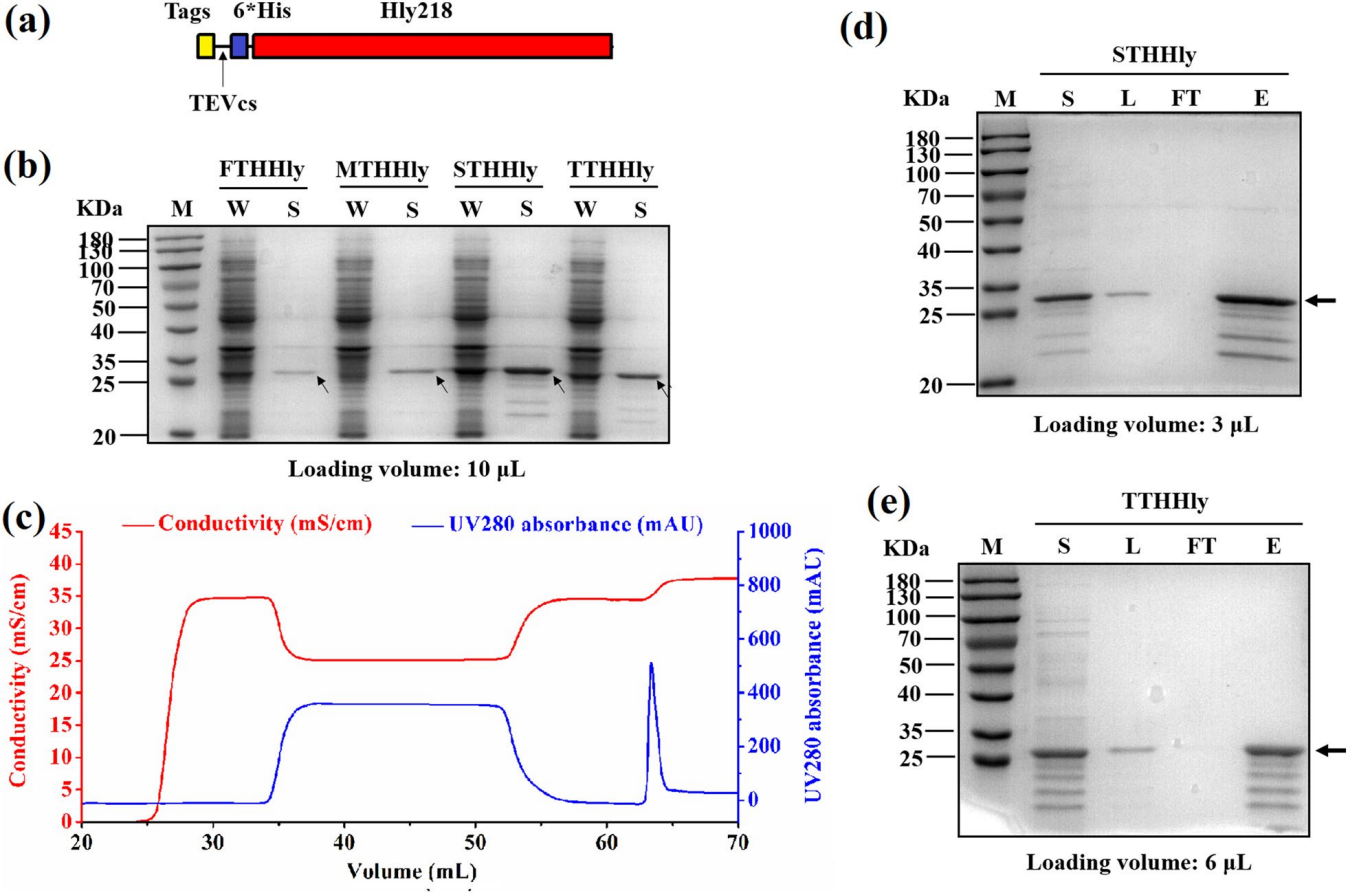
Secretory expression and purification of the tags fusion protein. **a** Plasmid pET-Tag-THHly. **b** Secretory expression of the Tag-THHly fusion proteins. *W* whole-cell lysate, *S* supernatant. **c** Purification curve of STHHly. **d**, **e** Purification of STHHly (**d**) and TTHHly (**e**). *S* supernatant, *L* loading sample, *FT* flow through, *E* eluent. The arrows indicate Tag-THHly in **b**, STHHly in **d**, and TTHHly in **e**

Based on the successful secretion of HTHHly, four other tag peptides, namely, Flag tag, Myc tag, S tag, and T7 tag, were also linked with the THHly to construct the fusion proteins (Fig. 4a). The four tag peptides were successfully secreted to supernatant with considerable efficiency (Fig. 4b). The yields of the fusion proteins in the supernatant were estimated to be 19.3 (FTHHly), 142.7 (HTHHly), 35.5 (MTHHly), 337 (STHHly), and 92.4 (TTHHly) mg/L according to the concentrations of the eluents. The secretory level as high as 337 mg/L at the cell density of OD_600_ = 2.52 demonstrate the great potential of the hemolysin system in the large-scale manufacturing of these tag peptides. Excessive heterogeneous proteins intracellularly accumulated in conventional expression systems probably affect the cell viability, thus the secretion of expressed protein to the supernatant could improve not only the yields but also the product quality, including biological activity, solubility, and stability (Feilmeier et al. 2000). In other secretion systems, the POI would first be secreted to the periplasm through the general secretory (Sec) or twin-arginine translocation (Tat) pathways, and subsequently traverse the outer membrane to the extracellular medium, thus described as a two-step process (Freudl 2018). However, the space of the periplasm is limited and protein accumulation may cause cell disruption (Linton et al. 2012). In addition, Sec pathway is critical for cellular viability, and Tat pathway can be indispensable depending on the strain and growth conditions (Natale et al. 2008). In comparison with them, the overload running of the Hly secretion systems didn’t show detectable side effects on the cell viability in our work.

The supernatants were obtained by centrifugation and the STHHly/TTHHly fusion proteins were purified by one-step nickel affinity chromatography (Fig. 4c, only the purification curve of STHHly is shown). The isolation and purification procedures for target proteins would be simplified and the cost markedly reduced, since there is no need for cell disruption, which usually leads to host cell derived contaminations. With the unwanted proteins removed, the fusion proteins were concentrated in the eluent (Fig. 4d, e). We also noticed that there were unexpected bands with lower molecular weights than the target proteins. Given that the degraded fragments can be captured by nickel column and were the same in STHHly and TTHHly, it is amenable to conclude that the fusion proteins were degraded at the C-terminal of Hly218. These bands can also be detected by anti-His tag antibody (Additional file 1: Fig. S1). The function domain of Hly218 for the secretory expression is primarily located in the last 50–60 amino acids (Yin et al. 1995), so the degradation probably occurred in the supernatant after secretion.

### Secondary purification of the tags and verification of the immunogenicity

**Fig. 5:**
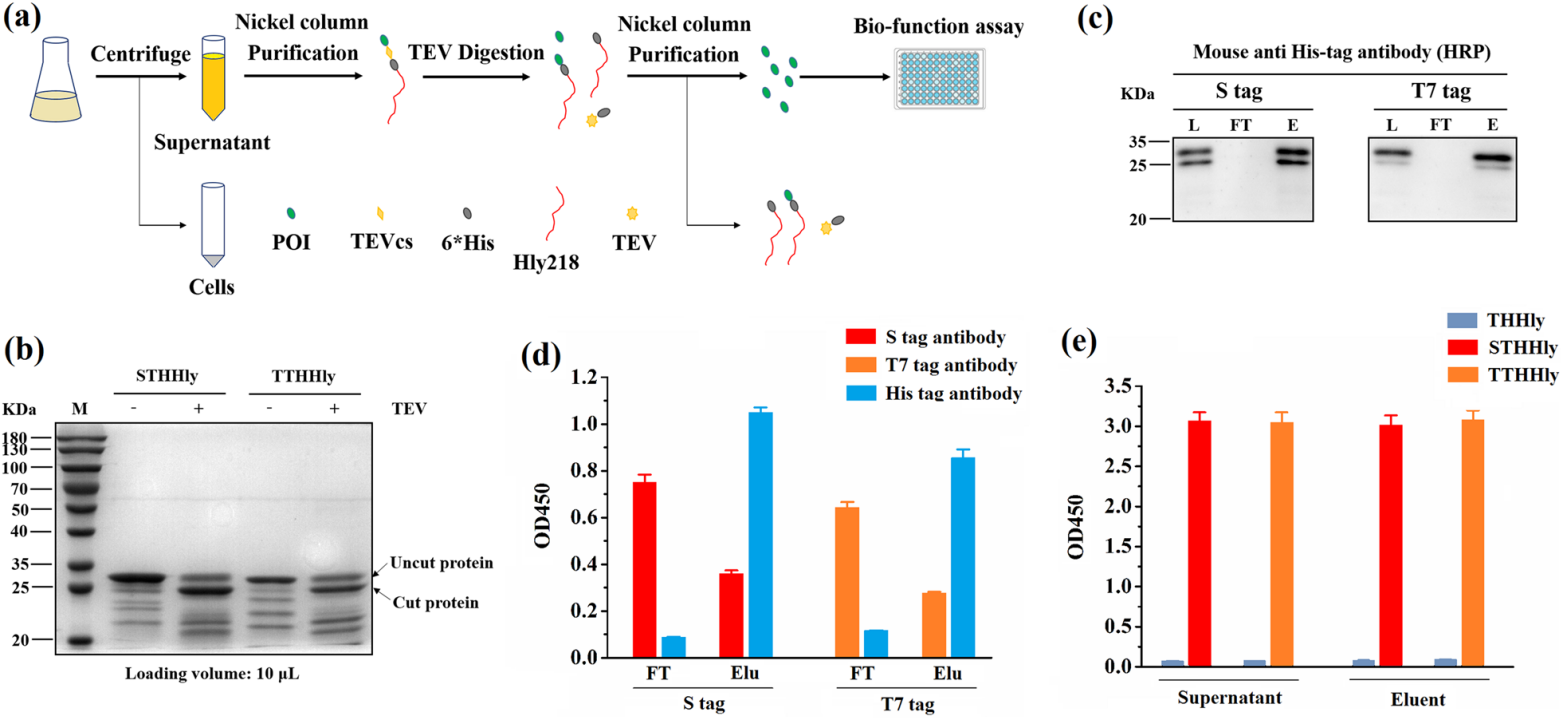
Secondary purification of the tags and verification of the immunogenicity. **a** Technical map of the purification procedure. **b** Digestion of STHHly and TTHHly with TEV enzyme. **c** Purification of S tag and T7 tag. *L* loading sample, *FT* flow through, *E* eluent. The samples were detected by a mouse anti-His tag antibody. **d**, **e** Verification of the immunogenicity of the tags in FT (**d**), eluent and supernatant (**e**). *Elu* eluent

The schematic illustration of purification is shown as Fig. 5a. The purified STHHly and TTHHly fusion proteins were digested by the TEV enzyme and the shift of bands after digestion was observed (Fig. 5b). The shift was also seen for the bands of the degraded fragments, which further proved the integrity of the POI-TEVcs-6*His domain. Since the HHlyX released from the fusion protein can also be captured by the nickel column, such a degradation is not problematic. The released S tag and T7 tag were collected in the flow through (FT) fraction and their immunogenicity was confirmed by ELISA assay (Fig. 5d). To rule out the possibility that a tiny amount of leaked STHHly/TTHHly would interfere with the immunogenicity test, His tag antibody was applied as negative control. The results show that by TEV cleavage and nickel affinity chromatography the tag peptides can be released from the fusion protein and displayed intrinsic immunogenicity. The design of the POI-THHly construct is reasonable for that the HHly218/HHlyX, the un-cut fusion proteins, and the His tag conjugated TEV enzyme can be removed by the nickel column, and only the tag peptides would be flowed through (Fig. 5a). Based on these, the hemolysin secretion system can be utilized to produce short peptides cost-effectively in comparison with conventional strategies, such as chemical synthesis.

To further explore the application potential of the THHly platform, the supernatants and the eluents of the first round purification, which contained the tag peptides in fusion form, were detected for the immunogenicity. Strong OD_450_ absorbance was observed for both the supernatants and the eluents of STHHly/TTHHly (Fig. 5e, THHly as the negative control). It illustrates that the THHly platform also can be applied to rapidly verify the interaction epitopes of antibodies, with high efficiency and lower cost.

### Secretory expression and purification of Cecropin A and Melittin

**Fig. 6:**
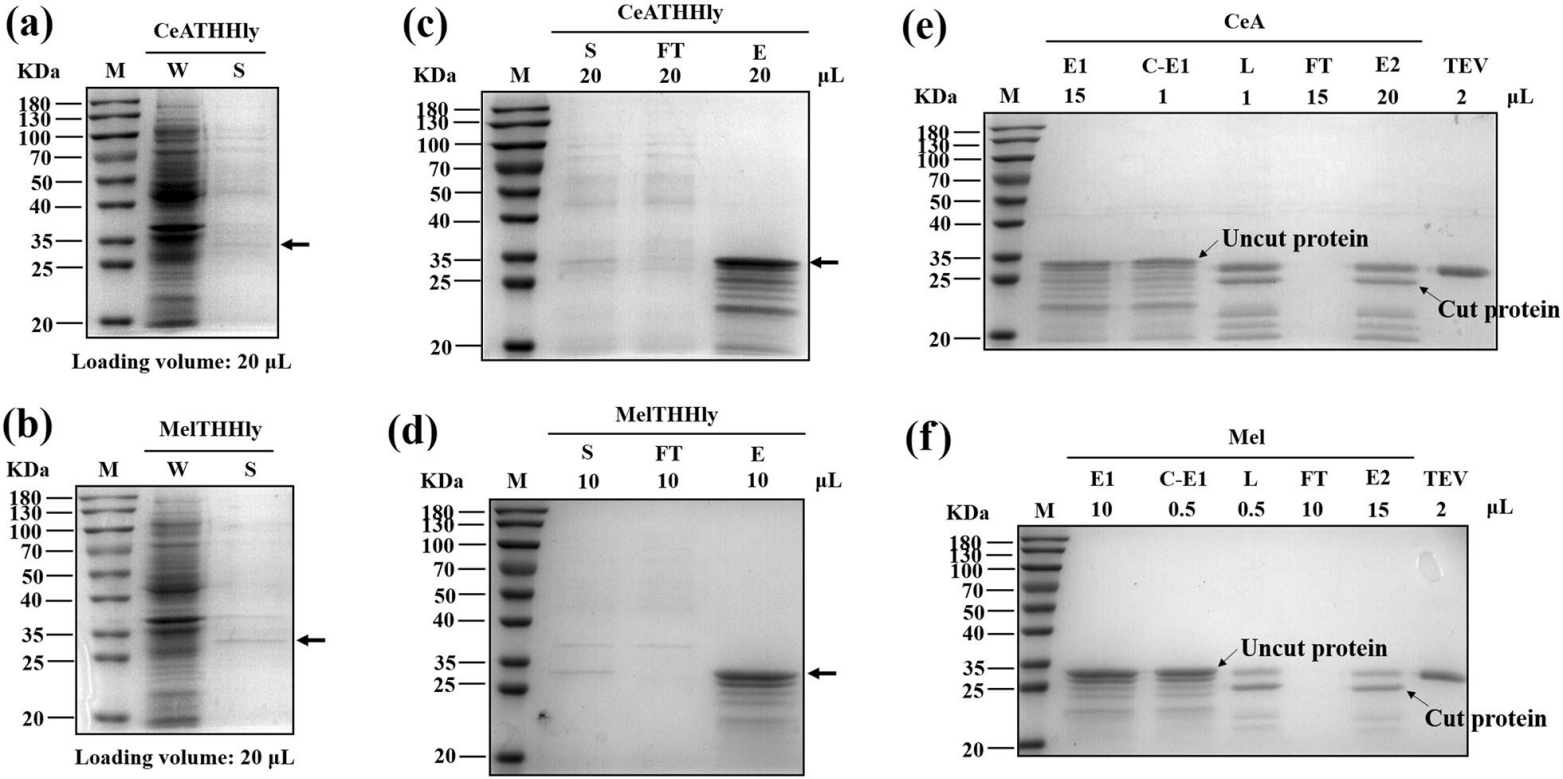
Secretory expression and purification of Cecropin A and Melittin. **a**, **b** Secretory expression of CeATHHly (**a**) and MelTHHly (**b**). *W* whole-cell lysate, *S* supernatant. **c**, **d** Purification of CeATHHly (**e**) and MelTHHly (**f**). *S* supernatant, *FT* flow through, *E* eluent. **e**, **f** Purification of CeA (**g**) and Mel (**h**). *C-E1* concentrate of eluent1. The arrows indicate CeATHHly in **a** and **c**, and MelTHHly in **b** and **d**

The results above confirm that it is practical to use *E. coli* to export POI-THHly fusions. We next investigated whether two AMPs, Cecropin A and Melittin, can be secreted and purified likewise. The bands corresponding to CeATHHly and MelTHHly were visible on the gel (Fig. 6a, b). The fusion proteins were purified with nickel column (Fig. 6c, d) and concentrated with a 3 KDa ultrafiltration tube. The concentrate was digested with TEV enzyme and then underwent the secondary purification step (Fig. 6e, f). The purification curves of CeATHHly and rCeA were also shown, as an example (Additional file 1: Fig. S2). The FT was applied to a 3 KDa ultrafiltration tube to concentrate the Cecropin A and Melittin. In summary, 0.39 mg Cecropin A and 0.88 mg Melittin can be obtained from one-liter medium (Tables 1 and 2). The yields are not as desired as the tag peptides, but the high secretory efficiency (no corresponding band was observed in the whole-cell lysate) implies that it was probably due to the intrinsic low expression property.

**Table 1:** Summary of the purification of Cecropin A

Purification step	Concentration (μg/mL)	Volume (mL)	Content (μg)	Recovery (%)	Overall recover (%)
Cell free supernatant	4.37	100	436.86	100	100
Nickel affinity chromatography	118.61	3.4	403.27	92.31	92.31
Sample for Ultrafiltration	118.61	1.5	177.92	–	–
Ultrafiltration	1603.7	0.1	160.37	90.14	83.21
Sample for cleavage	1603.7	0.08	128.29	–	–
CeA in the sample	260.06	0.08	20.8	–	–
Ultrafiltration	867.91	0.02	17.36	83.46	69.45

**Table 2:** Summary of the purification of Melittin

Purification step	Concentration (μg/mL)	Volume (mL)	Content (μg)	Recovery (%)	Overall recovery (%)
Cell free supernatant	16.39	100	1639.41	100	100
Nickel affinity chromatography	347.68	4.2	1455.63	88.79	88.79
Sample for ultrafiltration	347.68	2	695.36	–	–
Ultrafiltration	5984.22	0.1	598.42	86.06	76.41
Sample for cleavage	5984.22	0.08	478.74	–	–
Mel in the sample	755.9	0.08	60.47	–	–
Ultrafiltration	2091.5	0.02	41.83	69.17	52.85

To the best of our knowledge, until now there is few articles that have reported extracellular secretory expression of AMPs for the industrialized production using *E. coli* including one that exported CeA in *E. coli* by the curli fibers system (Wang et al. 2017). It is quite enlightening but indeed some problems hamper its wide applications which include: (1) necessary manipulations on the genome before transformation; (2) low secretory efficiency making the incubation time elongated to 3 days; (3) hardship to scale up to fermenter, since the curli fibers can only be formed in a static culture mode; (4) contamination by the host cell proteins for that when DTT mediated the self-cleavage of Mxe, it disrupted the cell membrane and caused cell lysis; (5) extra 42 amino acids at the N-terminal of POI. In this regard, our work laid a more solid foundation for the secretory expression of recombinant proteins in *E. coli*.

In the Hly secretion system, protein cargo must be either completely or partially unfolded to secrete successfully (Schwarz et al. 2012a, b; Kim et al. 2016). The primary drawback of using this system for protein production is a limited heterologous substrate range. Some studies related folding kinetics to “secretability” of the HlyA system and found that wild-type poorly secreted proteins were secreted at much higher levels when the slow-folding mutations were introduced (Bakkes et al. 2010), but this scheme is hard to be extended to all cases. As a type of proteins without tertiary structure, AMPs match this system well.

### Determination of the anti-microbial/anti-tumor activity of rCeA/rMel

**Fig. 7:**
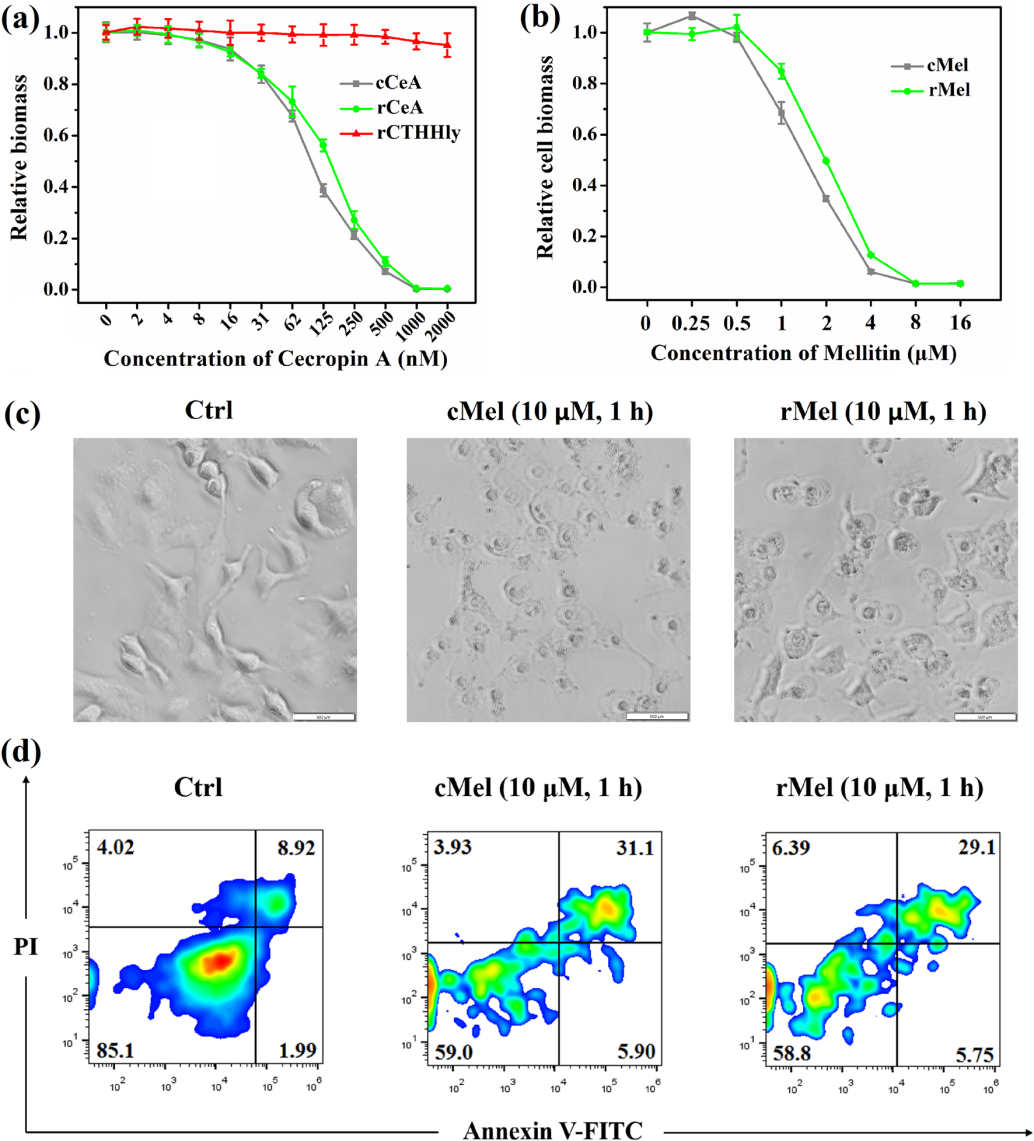
Determination of the anti-microbial/anti-tumor activity of rCeA/rMel. **a**, **b** Comparison of the anti-microbial activity of rCeA/cCeA (**a**) and anti-tumor activity of rMel/cMel (**b**). **c**, **d** Comparison of the tumor lysis capacity (**c**) and pro-apoptosis effects of rMel and cMel (**d**). *E. coli* 25,922 cells were incubated with different concentrations of cCeA and rCeA for 18 h, then the OD600 values were measured. A549 cells were incubated with different concentrations of cMel and rMel for 8 h, the numbers of living cells were detected with CCK8 reagent. For apoptosis detection, A549 cells were incubated with 10 μM melittin for 1 h, and subsequently labeled with Annexin V-FITC/PI and analyzed with a CytoFlex S

In this study, the Cecropin A and Melittin released from the fusion protein contained extra six C-terminal amino acid residues which remained after TEV enzyme cleavage. To verify whether the additional C-terminal amino acid residues would affect the anti-bacterial/anti-tumor activity, validation experiments were conducted to compare the recombinant Cecropin A (rCeA) and Melittin (rMel) produced in our lab with the commercial ones (cCeA and cMel). The minimum inhibitory concentration (MIC) of the rCeA turned out to be 1 μM (Fig. 7a), the same as that of cCeA. The IC_50_ of rMel to A549 was around 1.8 μM, also close to that of cMel, 1.4 μM (Fig. 7b). At 10 μM, rMel displayed similar tumor lysis capacity and pro-apoptosis effect (Fig. 7c, d) as cMel. The data demonstrate that the AMPs produced by the THHly system were of full bioactivities.

**Fig. 8:**
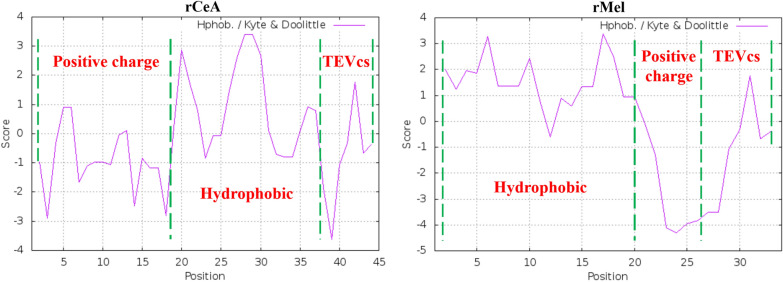
Charge and hydrophobicity profiles of recombinant rCeA and rMel analyzed by ExPASy. The amino acid sequences of rCeA and rMel were import into https://web.expasy.org/protscale/ and analyzed by Hphob/Kyte & Doolittle mode

As one of the earliest characterized AMPs, the mechanism by which CeA kills bacteria have been widely investigated. While the precise mechanism remains unveiled, there is a broad consensus that the anti-bacterial effect of CeA is attributed primarily to its ability to bind and assemble on the outer membrane, subsequently permeabilize and cause biofilm disruption (Rangarajan et al. 2013). Both the positive-charged N-terminal domain and hydrophobic C-terminal domain are essential for bio-activity (Nielsen et al. 2021). Such a structure–function relationship is also applicable to Mel, except that the positive-charged and hydrophobic amino acids distribute at the C and N-terminal, respectively (Jeon et al. 2019). We suggest that the extra six residues which possess negative charges and weak hydrophobicity disturb the hydrophobic interaction of CeA and neutralize the positive charges of Mel, thus having a small impact on the bioactivity (Fig. 8; https://web.expasy.org/protscale/). From this point of view, another advantage of the THHly platform is that the cells would be saved from the toxicity of AMPs, owing to the attached long tail “THHly” which blocked the anti-microbial activity (Fig. 7a).

## Conclusions

Many attempts have been made in the secretory expression of recombinant POI in *E. coli* over past decades but with little success. Based on the hemolysin secretion system of *E. coli*, a tandem fusion of POI, TEV enzyme cleavage site, 6*His tag and hemolysin signal sequence (Hly218) was designed in this study, namely, THHly. To explore whether the THHly system was generally applicable to produce different peptides products, five tag peptides and two AMPs were used as research models, and all of the fusion proteins were successfully secreted to the supernatant with different yields (4–337 mg/L). Attributed to the secretory expression, the downstream purification procedures were simple and cost-effective. First, the fusions were collected by centrifugation and nickel affinity chromatography. Then the POIs were released by TEV enzyme digestion and obtained by a secondary nickel affinity chromatography. The results of ELISA, MIC, and tumor-killing assays demonstrate that the recombinant tag peptides and AMPs possessed the intrinsic immunogenicity or the anti-microbial/anti-tumor properties.

Recombinant peptides synthesis in *E. coli* has been attaining large consideration for its obvious benefits in comparison with chemical synthesis, such as environment-friendly, cost-effective, and easy manipulations. The THHly system reported in this study is the first generalized platform for the secretory production of tag peptides and AMPs. The processes and techniques proposed here can facilitate the large-scale manufacturing of pharmaceutical peptides for pre-clinical or clinical studies. Meanwhile, it’s a meaningful attempt to achieve the secretory production of recombinant proteins in *E. coli*, which sheds light on the development of more effective platforms for the efficient production of larger molecules with complicated tertiary structures.

## Methods

### Plasmids, DNA constructs, and peptide sequences

Plasmids, DNA constructs, and peptide sequences used in this study are summarized in Table 3. The DNA sequences of HlyB–HlyD (UniProtKB: Q47258; P09986) and TEVcs-6*His-HlyA, which contain the last 218 amino acids of HlyA (UniProtKB: P09983) were chemically synthesized. The HlyB–HlyD was amplified by PCR and cloned into the pBAD-gIII B by Gibson Assembly to construct the plasmid pBAD-BD for the expression of the two accessory proteins. The sequences of tag proteins and AMPs were linked with THHly by PCR and cloned into the pET-28a by Gibson Assembly to construct the expression plasmids. All the constructs were verified by DNA sequencing.

**Table 3:** Summary of the plasmids, DNA constructs, and peptide sequences

Backbone	DNA constructs	Plasmids	Peptide sequences
pBAD-gIII B	HlyB–HlyD	pBAD-BD	–
pET-28a	TEVcs-6*His-HlyA	pET-THHly	–
	His tag-TEVcs-6*His-HlyA	pET-HTHHly	HHHHHH
	T7 tag-TEVcs-6*His-HlyA	pET-TTHHly	MASMTGGQQMG
	S tag-TEVcs-6*His-HlyA	pET-STHHly	KETAAAKFERQHMDS
	Flag tag-TEVcs-6*His-HlyA	pET-FTHHly	DYKDDDDK
	Myc tag-TEVcs-6*His-HlyA	pET-MTHHly	EQKLISEEDL
	Cecropin A-TEVcs-6*His-HlyA	pET-CeATHHly	KWKLFKKIEKVGQNIRDG IIKAGPAVAVVGQATQIAK
	Melittin-TEVcs-6*His-HlyA	pET-MelTHHly	GIGAVLKVLTTGLPALIS WIKRKRQQ

### Construction of the expression strains

The *E. coli* DH5α (Weidi, China) was used for plasmid amplification and the *E. coli* BL21 (DE3) (Weidi, China) was used for protein expression. The expression plasmid and pBAD-BD were co-transformed into BL21 (DE3) and selected by LB agar plate containing Ampicillin (100 μg/mL) and Kanamycin (10 μg/mL). The clones on the plates were picked and cultured in 5 mL LB medium containing Ampicillin (100 μg/mL) and Kanamycin (10 μg/mL) at 37 °C and 220 rpm. The plasmids were extracted and digested by *Eco* RV Quick Cut restriction enzyme (Takara, Japan), the clones which harbor the two plasmids were preserved as the expression strains.

### Secretory expression of the tag proteins and AMPs

The expression strains were grown overnight in LB containing Ampicillin (100 μg/mL) and Kanamycin (10 μg/mL) at 37 °C and 220 rpm. Then the cultures were inoculated with 1% inoculation to shake flask containing fresh LB with Ampicillin (100 μg/mL) and Kanamycin (10 μg/mL). Two hours later, 0.08% (w/v) Ara was added to induce the expression of HlyB and HlyD. Four hours after the addition of Ara, cell cultures were induced with 100 μM IPTG and incubated for another 4 h. The cultures were sampled and centrifuged at 12,000×*g* for 3 min, the harvested cell pellets were suspended in ddH_2_O to the original volume and all the samples were analyzed by non-reducing SDS-PAGE. For the optimization of the secretory expression, different concentrations of Ara (0.005–0.32%), IPTG (10–1000 μM), and different induction temperatures (16, 25, 37 °C) were tested.

### Purification of the tag peptides and AMPs

For the purification of tag peptides, the cultures were centrifuged at 12,000×*g* for 3 min. The supernatant was diluted with buffer A (50 mM NaH_2_PO_4_, 300 mM NaCl, pH 8.0) at a ratio of 1:4 and filtered through a 0.45 μm filter (Sangon, China). The solution was then loaded onto an EzFast Ni FF column (BestChrom, China) pre-equilibrated with buffer A with the AKTA Start Protein Purification System (GE Healthcare, USA). After washing the column with 5 column volumes (CV) of buffer A, the fusion protein was eluted with buffer B (50 mM NaH_2_PO_4_, 300 mM NaCl and 200 mM imidazole, pH 8.0). The loading solution, FT, eluent were analyzed by SDS-PAGE and the concentration of the fusion protein was measured by BCA Quantification Kit (Vazyme, China). The purification procedure of the two AMPs was the same except that the supernatants were loaded onto the column without any dilution.

The fusion proteins were digested by His tag conjugated TEV enzyme (Beyotime, China) at 4 °C overnight. The 100 μL digestion solution was diluted with buffer A to 5 mL and filtered through a 0.45 μm filter. The solution was then loaded onto an EzFast Ni FF column pre-equilibrated with buffer A with the AKTA Start Protein Purification System. The column was washed with 5 CVs of buffer A and then eluted with buffer B. The loading solution, FT, eluent were analyzed by non-reducing SDS-PAGE. The AMPs in the FT were concentrated to 15–20 μL with an ultrafiltration tube (3KD, 0.5 mL) (Millipore, German).

### ELISA experiments

The samples were diluted in PBS to a final volume of 100 μL and coated overnight at 4 °C in a 96-well ELISA plate (Corning, USA). After three times wash with PBST (200 μL/well), the plate was blocked with PBST + 5%BSA (200 μL/well) at 37 °C for 2 h. The plate was washed with PBST (200 μL/well) three times and the anti-tag mAb (1:10,000) (Beyotime, China) was added (100 μL/well). After incubation at 37 °C for 1 h, the plate was washed with PBST (200 μL/well) three times, and Horseradish Peroxidase (HRP)-conjugated Goat anti mouse mAb (1:10,000) (Beyotime, China) was added (100 μL/well). After incubation at 37 °C for another 1 h, the plate was washed with PBST (200 μL/well) three times and the reaction was finished using tetramethylbenzidine (TMB) (Solarbio, China) and stopped with H_2_SO_4_ (2 M). Using a Synergy LX multi-mode reader (BioTek, USA), the OD_450_ value were detected. Each sample in the assay was implemented in triplicate.

### Anti-microbial activity detection test

The anti-microbial activity of Cecropin A was measured by a broth micro-dilution method as previously reported (Wiegand et al. 2008). Overnight cultures of *E. coli* 25,922 were diluted with fresh Mueller–Hinton Broth (MHB) to OD 0.1 at 600 nm and then further diluted 1:100 with fresh MHB. Subsequently 100 μL bacterial suspension was added to each well. The commercial (Apeptides, China) and bio-produced Cecropin A were serially diluted into MHB medium and added into the 96-well plates to a final concentration of 2 to 2000 nM. The plate was incubated at 37 °C for 18 h and the OD_600_ values were measured with Synergy LX multi-mode reader. Broth with bacterial inoculum without AMP and broth alone were used as control groups in which the relative biomass was set as 1 and 0, respectively.

### Anti-tumor activity detection test

Ten thousand A549 cells were seeded in 96-well plates for 18 h to achieve log growth phase and then incubated with commercial or bio-produced melittin (0–16 μM) in triplicate for each condition. After 8 h incubation, 10 μL of CCK8 solution (FluoreScence, China) was added to each well of the plate and further incubated for 2 h. The absorbance was measured by Synergy LX multi-mode reader at a wavelength of 450 nm. Apoptosis was assessed after A549 cells were incubated with 10 μM melittin for 1 h. The cells were trypsinized, collected and washed in cold PBS. Then the cells were labeled with Annexin V-FITC/PI (Vazyme, China) and analyzed with a CytoFlex S (Beckman Coulter, USA). Quantitative analysis of apoptotic cells was carried out using BD Flowjo VX software.

### Supplementary Information

**Additional file 1: Figure S1.** The detection of the degraded fragments by anti-His tag antibody. **Figure S2.** The purification curves of CeATHHly (a) and CeA (b).

## Data Availability

All data generated or analyzed during this study are included in this published article.

## Abbreviations

HlyA: Hemolysin A
AMPs: Anti-microbial peptides
TEV: Tobacco Etch Virus
POI: Protein of interest
T1SS: Type I secretion system
ABC: ATP-binding cassette
ELISA: Enzyme Linked Immunosorbent Assay
KDa: Kilo-Dalton
SDS-PAGE: Sodium dodecyl sulfate polyacrylamide gel electrophoresis
OD_600_: Optical density at a wavelength of 600 nm
Ara: Arabinose
IPTG: Isopropyl-beta-d-thiogalactopyranoside
CV: Column volumes
FT: Flow through
ATP: Adenosine–triphosphate
CeA: Cecropin A
Mel: Melittin
DTT: Dithiothreitol
MIC: Minimum inhibitory concentration
MHB: Mueller–Hinton Broth
BSA: Bovine serum albumin
PBS: Phosphate buffered saline
PBST: Phosphate buffered saline with Tween
