# Supplementary material for: A hemolysin secretion pathway-based novel secretory expression platform for efficient manufacturing of tag peptides and anti-microbial peptides in Escherichia coli
Source: Bioresour Bioprocess. 2021 Nov 26;8(1):115. doi: 10.1186/s40643-021-00471-6 (PMC10992379; doi:10.1186/s40643-021-00471-6)
Supplement: Supplementary file 1 — Additional file 1: Figure S1. The detection of the degraded fragments by anti-His tag antibody. Figure S2. The purification curves of CeATHHly (a) and CeA (b). [file 40643_2021_471_MOESM1_ESM.docx]

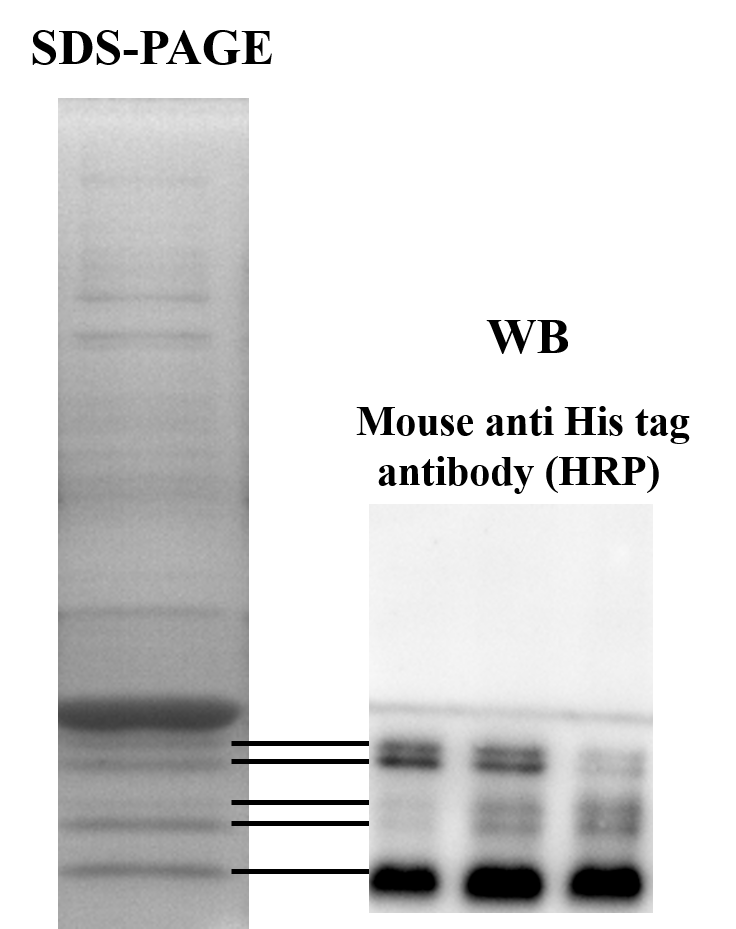


**Fig S1.** The detection of the degraded fragments by anti-His tag antibody


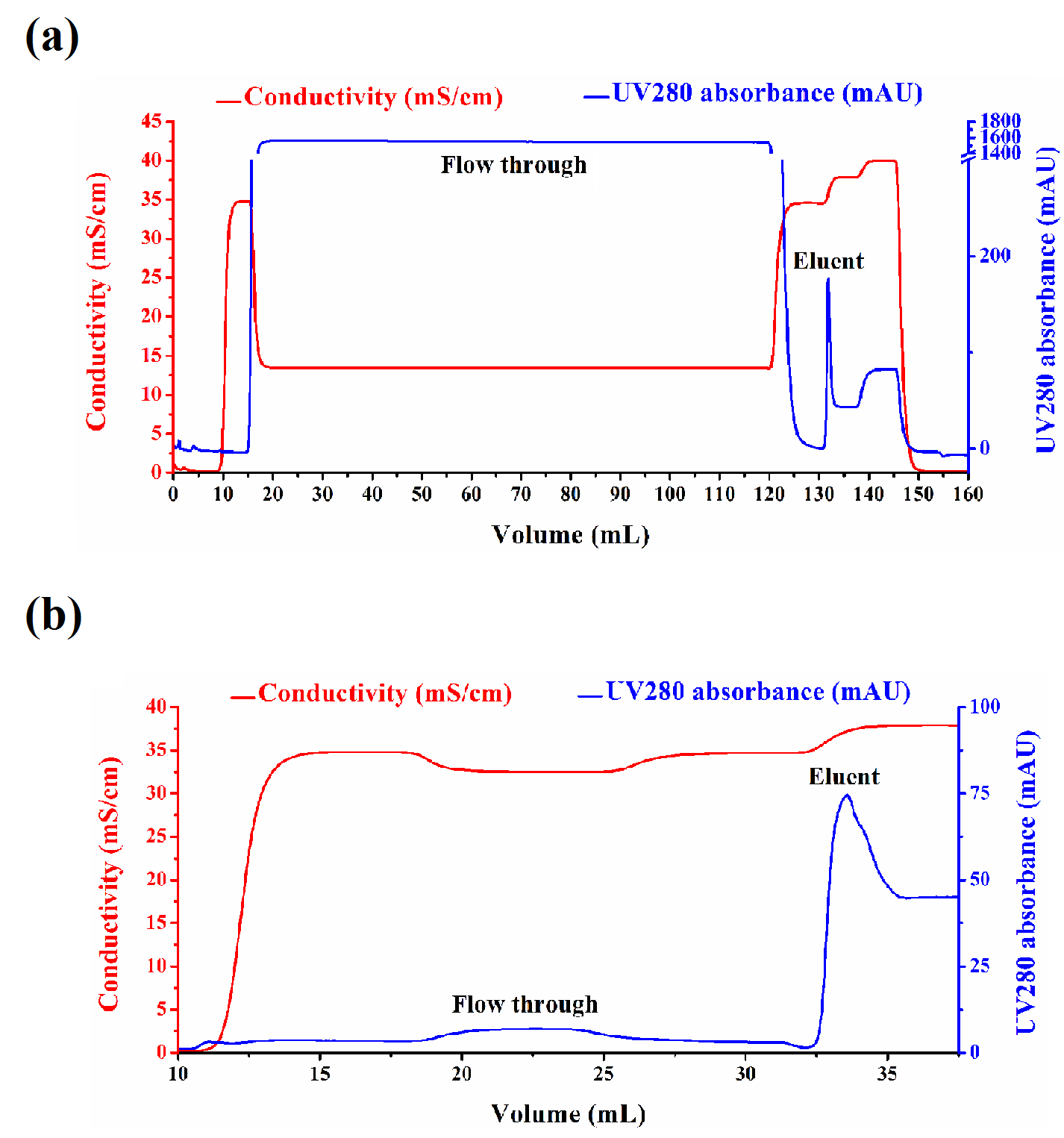


**Fig S2.** The purification curves of CeATHHly (a) and CeA (b)
